# Diagnostic and Treatment-Related Challenges in Sinonasal Teratocarcinosarcoma: A Report of Three Cases

**DOI:** 10.1155/2023/4788617

**Published:** 2023-11-11

**Authors:** W. F. Julius Scheurleer, Weibel W. Braunius, Bernard M. Tijink, Luuk M. Janssen, Frank A. Pameijer, Gerben E. Breimer, Ernst J. Smid, Remco de Bree, Lot A. Devriese, Johannes A. Rijken

**Affiliations:** ^1^Department of Head and Neck Surgical Oncology, University Medical Center Utrecht, Utrecht, Netherlands; ^2^Department of Radiology, University Medical Center Utrecht, Utrecht, Netherlands; ^3^Department of Pathology, University Medical Center Utrecht, Utrecht, Netherlands; ^4^Department of Radiation Oncology, University Medical Center Utrecht, Utrecht, Netherlands; ^5^Department of Medical Oncology, University Medical Center Utrecht, Utrecht, Netherlands

## Abstract

**Background:**

Sinonasal teratocarcinosarcoma is a rare, aggressive malignancy located almost exclusively in the nasal cavity, paranasal sinuses, or anterior skull base. Histopathological diagnosis can be challenging due to the heterogeneous composition.

**Methods:**

Retrospective analysis of 3 patients with sinonasal teratocarcinosarcoma diagnosed and treated at the University Medical Center Utrecht was conducted.

**Results:**

Patients presented with nasal obstruction, epistaxis, headaches, or behavioral changes. All three patients had locally advanced disease, and one had lymph node metastases. Two patients underwent surgery followed by radiotherapy, and one underwent neoadjuvant chemotherapy followed by surgery. The follow-up duration ranged from 3 to 32 months. All three patients died due to progression of their disease.

**Conclusion:**

Sinonasal teratocarcinosarcoma is characterized by rapid, aggressive local expansion. The prognosis is poor due to a high risk of metastases and locally recurrent disease. Multimodality treatment consisting of surgery, followed by (chemo)‐radiotherapy, is essential for optimizing outcomes. Neoadjuvant therapy offers a promising treatment option.

## 1. Introduction

Teratocarcinosarcoma is a malignant tumor localized almost exclusively in the nasal cavity, paranasal sinuses, and anterior skull base [1–4]. The vast majority of patients with sinonasal teratocarcinosarcoma (TCS) are male and in the fifth decade of life at the time of diagnosis [5]. TCS is characterized by rapid, remarkably aggressive growth with invasion of surrounding tissues [6]. The first description of these malignancies was supplied in 1983 by Shanmugaratnam et al., but the name was coined a year later by Heffner and Hyams [7, 8]. The World Health Organization (WHO) provided a distinction between TCS and other germ cell tumors in 2005 [9]. Yet, histopathologic diagnosis of TCS can be challenging due to its heterogeneous composition with the presence of primitive neuroepithelial elements and various epithelial and mesenchymal components (e.g., the cartilage, bone, or smooth muscle) [8]. Biopsies can be deceptive due to the morphological overlap of individual components with other sinonasal tumors such as olfactory neuroblastoma or squamous cell carcinoma, increasing the likelihood of misdiagnosis on biopsy [10]. Recently, molecular signatures were described in TCS, specifically biallelic inactivation of *SMARCA4* and activating *CTNNB1* mutation, which can immunohistochemically be revealed by SMARCA4 (BRG1) loss and aberrant nuclear ß-catenin expression, respectively [11, 12].

Because of its rarity, a consensus is lacking regarding the optimal management of TCS. Current treatment strategies have been based on limited case series and other retrospective studies [6]. Surgery remains the cornerstone of treatment [13]. Gross total resection is difficult to achieve because of the limited anatomical space for optimal surgical margins due to the proximity to vital structures [6]. Therefore, adjuvant (chemo)radiotherapy is often required [13]. Neoadjuvant systemic therapy in the treatment of TCS is a topic of debate and, as of yet, has been scarcely utilized [14]. In this case series, we present three patients with TCS treated at the University Medical Center Utrecht (UMCU) in the Netherlands.

## 2. Methods

In this retrospective single center study, the records of all patients with sinonasal malignancies were assessed, who were treated in UMCU, a tertiary referral center for head and neck malignancies, between January 2011 and June 2021. Clinical characteristics including sex, age at the time of diagnosis, medical history, tumor location, disease staging, treatment strategies, follow-up duration, and outcome were documented. Age at diagnosis was defined on the date of histopathologic confirmation. The duration of follow-up was calculated from the date of diagnosis to the last moment of follow-up or date of death. Histopathological nomenclature and classification are in accordance with the WHO definition of head and neck tumors and the 8^th^ edition of the TNM classification by the Union for International Cancer Control [15, 16]. Tumors staged according to previous editions of the TNM classification were restaged per the 8^th^ edition. Imaging consisted of magnetic resonance (MR) imaging, computed tomography (CT), chest X-ray, neck ultrasound, and (if applicable) fine needle aspiration cytology of suspected lymph nodes. The following antibodies were used: SMARCA4 (clone EPR3912; Abcam, Cambridge, MA, USA), ß-catenin (clone 14; Cell Marque, Austin, TX, USA), and SALL4 (clone 6E3; Cell Marque, Austin, TX, USA) on the Ventana BenchMark Ultra platform (Ventana Medical Systems, Tucson, AZ, USA). SALL4 was retrospectively performed on the cases as per Compton et al. [17]. SMARCA4 and beta-catenin were retrospectively performed on cases as they were diagnosed before the publications by Rooper et al. [11, 12] All patients were reviewed by the UMCU multidisciplinary head and neck oncology team (MDT) before treatment.

The authors declare that all procedures performed in studies involving human participants were in accordance with the ethical standards of the institutional research committee and with the 1964 Helsinki Declaration. For this type of study, formal consent is not required.

## 3. Results

Within a total cohort of 246 patients with sinonasal malignancies who were treated over a 10-year period, three patients with TCS were identified. The clinical characteristics of these three patients are summarized in Table 1.

### 3.1. Patient No. 1

**Figure 1:**
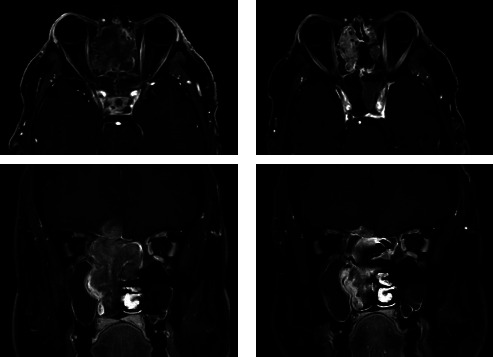
Axial and coronal postcontrast T1-weighted with fat saturation MR images of patient no. 1 with T4bN0M0 TCS located in the right nasal cavity and paranasal sinuses with contralateral extension. Note: cranial extension through the cribriform plate with (limited) invasion of brain parenchyma. (a): prior to neoadjuvant chemotherapy; (b): after 2 cycles of carboplatin/etoposide.

**Figure 2:**
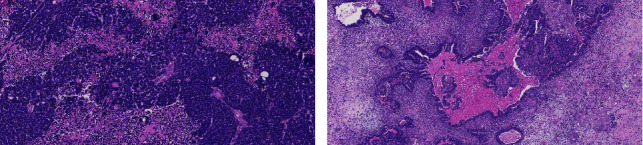
Histopathological images of patient no. 1. (a): undifferentiated component. (b): adenocarcinoma, squamous cell carcinoma, and sarcoma components.

A 56-year-old male presented with unilateral right-sided nose obstruction, epistaxis, progressive headaches, and a single instance of temporary vision loss two weeks prior. He had a medical history of stroke and myocardial infarction, for which he had been treated with coronary artery bypass grafts. Physical examination showed proptosis of his right eye and a polypoid mass in his right nasal cavity. Subsequent CT and MR imaging of the head and neck revealed a destructive mass laterally in the right nasal cavity and ethmoid sinus, extending caudally to the inferior nasal turbinate and posteriorly to the nasopharynx. Cranially, the tumor extended to the orbit and the anterior skull base with destruction of the cribriform plate and invasion of brain parenchyma (Figure 1). The biopsy only showed primitive neuroepithelial elements without immunohistochemical loss of SMARCA4 or aberrant nuclear ß-catenin expression. There was no sustentacular pattern in S100. There were overexpression of P16 and loss of Rb expression; however, no keratin expression was found. Either olfactory neuroblastoma (Hyams grade 4) or small cell neuroendocrine carcinoma was considered, and olfactory neuroblastoma was favored. The tumor was clinically staged as Kadish C olfactory neuroblastoma. After discussion in the MDT, the patient was scheduled for two neoadjuvant courses of chemotherapy (carboplatin and etoposide), to which he had a partial response as evaluated by MRI. Subsequently, the patient underwent a combined endonasal and transcranial tumor resection with reconstruction of the anterior skull base with a galea flap and titanium mesh. Histological evaluation of the resection specimen showed a malignant neoplasm with epithelial, mesenchymal, and primitive neuroepithelial elements. (Figure 2). SALL4 showed moderate positivity (2+) 50–70% of the primitive component and moderate-to-strong staining (2+ to 3+) in approximately 50% of adenocarcinoma components. Hence, the diagnosis was revised to ypT4b cN0M0 TCS. Postoperatively, the patient developed cerebrospinal fluid (CSF) leakage, for which he was treated with an external CSF drain and antibiotics. New postoperative imaging of the head and neck revealed leptomeningeal metastases, which in retrospect, although smaller, turned out to have already been present prior to treatment. Adjuvant chemoradiotherapy was cancelled, and the patient received best supportive care (BSC) until his death, three months after disease diagnosis.

### 3.2. Patient No. 2

**Figure 3:**
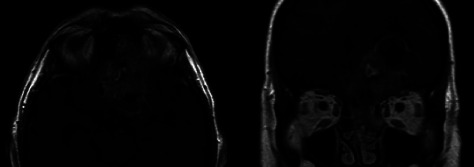
Axial and coronal postcontrast T1-weighted MR images of patient no. 2 with a T4bN0M0 tumor located in the left ethmoid sinus with gross intracranial extension. Note: extensive peritumoral (low signal intensity) edema resulting in a midline shift towards the right side.

A 70-year-old male presented with sudden cognitive deterioration and behavioral changes. He had a medical history of hypertension, hypercholesterolemia, and stroke. Physical examination showed a mass in the roof of the left nasal cavity. Subsequent CT and MR imaging of the head and neck revealed a mass in the left ethmoid sinus (Figure 3). The tumor extended cranially towards the anterior skull base, brain parenchyma, and left ventricle, resulting in a midline shift. The tumor was clinically staged as T4bN0M0. The patient underwent a combined endonasal and transcranial tumor resection with a galea flap and a titanium-mesh reconstruction of the anterior skull base, followed by adjuvant radiotherapy (60 Gy). Fifteen months after surgery, routine imaging revealed locoregional recurrent disease for which he received reirradiation (21 Gy). Thirteen months later, additional imaging revealed spinal leptomeningeal metastases for which he received palliative radiotherapy (20 Gy) and BSC. The patient died 32 months after diagnosis.

### 3.3. Patient No. 3

**Figure 4:**
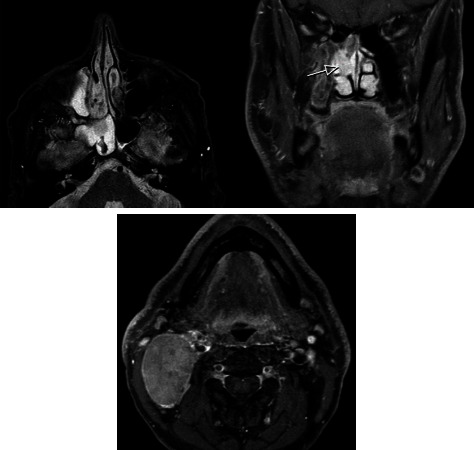
Axial STIR, coronal, and axial postcontrast T1-weighted with fat saturation MR images of patient no. 3 with a T3N2bM0 tumor located in the right nasal cavity and sphenoid sinus. On the axial image, the primary tumor has an intermediate signal intensity. Note: due to obstruction by the tumor, the right maxillary sinus and the sphenoid sinus are filled with retained secretions displaying high signal intensity. The coronal image shows the enhancing tumor in the right nasal cavity just medial to the middle turbinate (arrow). Axial postcontrast image lower in the neck with a markedly enlarged lymph node in level II (fine needle aspiration cytology: TCS lymph node metastasis).

**Figure 5:**
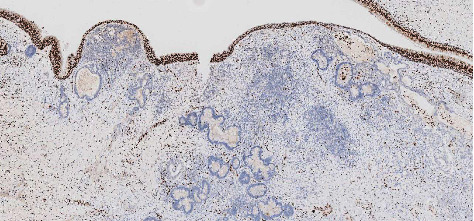
Histopathological images of patient no 3. Immunohistochemistry demonstrating loss of SMARCA4 expression in tumor cells of a patient with a teratocarcinosarcoma. Note: the positive internal control: expression of SMARCA4 in the superficial epithelium.

A 45-year-old male presented with unilateral right-sided nose obstruction and recurrent epistaxis. He had no relevant medical history. Physical examination showed a polypoid mass in the right ethmoid sinus and an enlarged lymph node on the left side of the neck (level IIa). Subsequent CT and MR imaging of the head and neck revealed a mass in the right ethmoid sinus, nasal cavity, and sphenoid sinus (Figure 4). Fine needle aspiration cytology of the neck mass showed lymph node metastases of TCS. The tumor was staged as T3N2bM0. The patient underwent endonasal endoscopic tumor resection and ipsilateral neck dissection (levels I-IV), followed by adjuvant radiotherapy (50 Gy). The SMARCA4 stain showed a loss of expression in tumor cells (Figure 5). SALL4 showed moderate-to-strong expression (2+ to 3+) in approximately 50% of the primitive neuroepithelial and adenocarcinoma components. ß-Catenin did not show aberrant nuclear expression. During follow-up, five months after surgery, the patient developed pulmonary metastases for which he received BSC. Unfortunately, the patient died six months after diagnosis due to progression of the disease.

## 4. Discussion

This single-center case series describes three male patients, between 45 and 70 years of age, with TCS treated in a tertiary referral center. These tumors are exceptionally rare and aggressive, resulting in poor survival rates. Available literature about TCS is limited, consisting mainly of individual case reports or case series. TCS patients are typically middle aged, although their occurrence has also been observed in children [5, 6, 8]. Similar to other malignancies of the head and neck, there is a predisposition in men. However, this disparity is seemingly more pronounced in TCS, with male patients constituting nearly 90% of cases in some studies [1]. The underlying cause for this male predominance is currently unknown. It may result from inherent biological differences between men and women, or a difference in exposition to potentially malignant agents, but this has not yet been explored. No other risk factors or tumor predisposition syndrome associations have been described [16]. Besides the nose, paranasal sinuses, and anterior skull base, occurrence of TCS in the nasopharynx, oral cavity, and thyroid gland has also been reported. However, these likely constitute separate entities, possibly with different progenitor cells [1–4].

Typical symptoms for locally advanced sinonasal malignancies are unilateral nose obstruction and recurrent epistaxis as was the case for two of our patients. In addition, TCS patients are prone to complaints related to intracranial extension, such as headaches and neurological symptoms (i.e. cognitive and behavioral changes). These symptoms are in line with those described in previous literature. Misra et al. reported nasal obstruction and epistaxis as the most frequently occurring primary symptoms in TCS (75.6% and 62.8%, respectively), followed by frontal headaches (19.8%) [5].

Histopathological confirmation can be complex as TCS displays a high degree of heterogeneity with an admixture of epithelial, mesenchymal, and primitive neuroepithelial elements. Hence, inadequate biopsy samples are at risk of misdiagnosis [10]. For example, if only primitive neuroepithelial elements are sampled, olfactory neuroblastoma or small cell neuroendocrine carcinoma can be considered. To further complicate differential diagnosis, recently, olfactory carcinoma and teratocarcinosarcoma-like NAB2: STAT6 fusion-related neoplasms have been described [18, 19].

Diagnosis requires a combination of malignant (neuro) epithelial and mesenchymal components, but TCS is also known to display benign components [8]. Most cases harbor clear “fetal-type” squamous elements comprising squamous cells with clear cytoplasm, as these are observed in roughly 50%–75% of tumors [8, 20]. Chemotherapy-induced maturation in the neuroectodermal component within TCS has also been described in this disease [21]. Immunohistochemistry often shows expression compatible with various elements (such as CAM5.2 in adenocarcinoma components or P40 in squamous elements) [22–24]. Recent advances in diagnosing TCS are using SMARCA4 (BRG1) immunohistochemistry which shows loss of expression in approximately 70% of cases and aberrant nuclear ß-catenin expression in a subset of cases [11, 12]. This loss of expression suggests that TCS is part of a spectrum of SMARCA4-deficient sinonasal carcinomas and could benefit from similar novel targeted therapies [25]. Such therapies include enhancer of zeste homolog 2 (EZH2), Aurora kinase A (AURKA), and inhibitors of cyclin-dependent kinase 4/6 (CDK4/6) [26–28]. However, aberrant ß-catenin expression could not be found by other groups [17, 29]. We found one case with SMARCA4 loss and no cases with nuclear ß-catenin expression. Furthermore, SALL4 expression was found in two cases.

Management of TCS is challenging due to the tumor's aggressive local expansion. Furthermore, these tumors have the potential to metastasize to both regional lymph nodes and distant sites, such as the lungs, bones, and leptomeninges [17, 30, 31]. In order to achieve the best possible clinical outcome, an assertive multimodality treatment approach is essential. The current preferred treatment method consists of surgical resection followed by radiotherapy, as was the case for patients 2 and 3 [5, 6]. However, because of the rarity of TCS, current management strategies are based entirely on case series and limited retrospective studies. All patients developed either locoregional recurrent disease or distant metastases and eventually died of their disease. This exemplifies how common recurrent disease is in TCS patients, with a recurrence rate of approximately 38% and a mean time to recurrence of 19.5 months [6, 32]. As a consequence, TCS is associated with a high mortality rate. Chapurin et al. reported a mean 2-year survival rate of 55% in all patients [6]. Similarly, Misra et al. reported a rate of survival of 56.5% for patients who were treated with a combination of surgery and radiotherapy at an average follow-up of 45.4 months [5].

As Modeto et al. reported high response rates after administering neoadjuvant chemotherapy in patients with olfactory neuroblastoma, one of our patients had been treated with neoadjuvant chemotherapy due to the extension of disease (brain parenchyma) and discussion pending definitive diagnosis after biopsy (either small cell neuroendocrine carcinoma or olfactory neuroblastoma) [33]. He subsequentially underwent swift surgical resection, but unfortunately, the patient developed leptomeningeal metastases within weeks. Systemic therapy is currently utilized in only a small fraction of TCS patients but is associated with the highest survival rate and lowest recurrence rate [5, 6]. Furthermore, neoadjuvant chemotherapy has proven effective in similarly aggressive sinonasal malignancies such as sinonasal undifferentiated carcinoma (SNUC). It is important to note that (neoadjuvant) chemotherapy may affect the immune system (i.e. neutropenia), resulting in increased perioperative risk, with postponement of surgery as a possible consequence. The preferred treatment after a partial response to neoadjuvant therapy is also not yet clear. Amit et al. reported improved survival for definitive chemoradiotherapy compared to surgery in SNUC patients who had a partial or complete response to neoadjuvant chemotherapy. In contrast, the same study reported improved survival for surgery plus (chemo) radiotherapy compared to definitive chemoradiotherapy in a group of patients who had no response to neoadjuvant chemotherapy [34]. The fact that patient 1 showed a (partial) local response to neoadjuvant therapy indicates that this treatment may be a valuable addition to the treatment options for this disease with a poor prognosis, thus warranting the exploration of novel therapeutics.

## 5. Conclusion

Sinonasal teratocarcinosarcomas are rare, aggressive tumors. Histopathological confirmation of diagnosis can be challenging. Multimodality treatment consisting of surgery, followed by radiotherapy with or without chemotherapy, is essential for optimizing oncological outcomes. Nevertheless, patients have a poor prognosis, and the risk of metastases and locally recurrent disease is high. Neoadjuvant therapy, whether followed by surgery or definitive chemoradiotherapy, offers a promising treatment option as displayed in one of our patients. In order to further investigate this and develop a better understanding of this malignancy, collaborative research efforts are required.

## Figures and Tables

**Table 1:** Patient characteristics.

Patient no	Sex	Age at diagnosis^*∗*^	Tumor localization	Staging at diagnosis^*∗∗*^	Treatment strategy	Recurrent disease	Time to recurrent disease (months)^*∗∗∗*^	Recurrent disease treatment	Follow-upduration (months)	Outcome
1	Male	56	Nasal cavity, right	T4bN0M0	CT + S	Leptomeningeal metastases	0	BSC	3	DOD
2	Male	70	Ethmoid sinus, left	T4bN0M0	S + RT	Locoregional; leptomeningeal metastases^*∗∗∗∗*^	15; 28	Radiotherapy; radiotherapy	32	DOD
3	Male	45	Nasal cavity, right	T3N2bM0	S + RT	Pulmonary metastases	5	BSC	6	DOD

^*∗*^Age at diagnosis determined on the date of histopathologic confirmation. ^*∗∗*^Staged in accordance with the TNM 8^th^ edition; soft tissues; head and neck. ^*∗∗∗*^Defined as time since initial treatment. ^*∗∗∗∗*^Patient no. 2 first developed locoregional recurrent disease. Leptomeningeal metastases occurred at a later stage. CT = chemotherapy; S = surgery; RT = radiotherapy; BSC = best supportive care; DOD = death of disease.

## Data Availability

The data used to support the findings of this study are available from the corresponding author upon reasonable request.

## References

[1] Agaimy A., Witkowski L., Stoehr R. (2020). Malignant teratoid tumor of the thyroid gland: an aggressive primitive multiphenotypic malignancy showing organotypical elements and frequent DICER1 alterations-is the term “thyroblastoma” more appropriate?. *Virchows Archiv*.

[2] Rotenberg B., El-Hakim H., Lodha A., MacCormick A., Ngan B. Y., Forte V. (2002). Nasopharyngeal teratocarcinosarcoma. *International Journal of Pediatric Otorhinolaryngology*.

[3] Crazzolara R., Puelacher W., Ninkovic M. (2004). Teratocarcinosarcoma of the oral cavity. *Pediatric Blood and Cancer*.

[4] Carrizo F., Pineda-Daboin K., Neto A. G., Luna M. A. (2006). Pharyngeal teratocarcinosarcoma: review of the literature and report of two cases. *Annals of Diagnostic Pathology*.

[5] Misra P., Husain Q., Svider P. F., Sanghvi S., Liu J. K., Eloy J. A. (2014). Management of sinonasal teratocarcinosarcoma: a systematic review. *American Journal of Otolaryngology*.

[6] Chapurin N., Totten D. J., Morse J. C. (2021). Treatment of sinonasal teratocarcinosarcoma: a systematic review and survival analysis. *American Journal of Rhinology and Allergy*.

[7] Shanmugaratnam K., Kunaratnam N., Chia K. B., Chiang G. S., Sinniah R. (1983). Teratoid carcinosarcoma of the paranasal sinuses. *Pathology*.

[8] Heffner D. K., Hyams V. J. (1984). Teratocarcinosarcoma (malignant teratoma?) of the nasal cavity and paranasal sinuses A clinicopathologic study of 20 cases. *Cancer*.

[9] Cardesa A., Luna M. A., Barnes L., Eveson J. W., Reichart P., Sidransky D. (2005). Germ cell tumours. *World Health Organization Classification of Tumours. Pathology and Gen*.

[10] Sable M., Kakkar A., Garg K., Suri V. (2017). Sinonasal teratocarcinosarcoma: an underdiagnosed entity posing diagnostic challenges. *Turkish Neurosurgery*.

[11] Rooper L. M., Uddin N., Gagan J. (2020). Recurrent loss of SMARCA4 in sinonasal teratocarcinosarcoma. *The American Journal of Surgical Pathology*.

[12] Yanik M., Scott M., Bradford C. (2017). Pathogenetic analysis of sinonasal teratocarcinosarcomas reveal actionable *β*-catenin overexpression and a *β*-catenin mutation. *Journal of Neurological Surgery Part B: Skull Base*.

[13] Budrukkar A., Agarwal J. P., Kane S. (2010). Management and clinical outcome of sinonasal teratocarcinosarcoma: single institution experience. *Journal of Laryngology and Otology*.

[14] Prabhash K., Joshi A., Noronha V. (2015). Neoadjuvant chemotherapy in advanced sinonasal teratocarcinosarcoma with intracranial extension: report of two cases with literature review. *Journal of Cancer Research and Therapeutics*.

[15] Brierley J. D., Gospodarowicz M. K., Wittekind C. (2016). *The TNM Classification of Malignant Tumours*.

[16] Bishop J. A., Loney E. L., Thompson L. D. R., Board W. C. T. E. (2022). Nasal cavity, paranasal sinuses, and skull base tumours. *Head and Neck Tumours*.

[17] Compton M. L., Lewis J. S., Faquin W. C., Cipriani N. A., Shi Q., Ely K. A. (2022). SALL-4 and beta-catenin expression in sinonasal teratocarcinosarcoma. *Head and Neck Pathology*.

[18] Rooper L. M., Bishop J. A., Faquin W. C. (2022). Sinonasal tumors with neuroepithelial differentiation (olfactory carcinoma): delineation of their pathologic and clinical features with insights into their relationship to olfactory neuroblastoma and sinonasal carcinoma. *The American Journal of Surgical Pathology*.

[19] Stevens T. M., Rooper L. M., Bacchi C. E. (2022). Teratocarcinosarcoma-like and adamantinoma-like head and neck neoplasms harboring NAB2::STAT6: unusual variants of solitary fibrous tumor or novel tumor entities?. *Head and Neck Pathology*.

[20] Wei S., Carroll W., Lazenby A., Bell W., Lopez R., Said-Al-Naief N. (2008). Sinonasal teratocarcinosarcoma: report of a case with review of literature and treatment outcome. *Annals of Diagnostic Pathology*.

[21] Kane S. V., Karpate A. A., Bal M., Juvekar S. L., Pai P. S. (2009). Chemotherapy-induced neuronal maturation in sinonasal teratocarcinosarcoma--a unique observation. *Head and Neck Pathology*.

[22] Pai S. A., Naresh K. N., Masih K., Ramarao C., Borges A. M. (1998). Teratocarcinosarcoma of the paranasal sinuses: a clinicopathologic and immunohistochemical study. *Human Pathology*.

[23] Endo H., Hirose T., Kuwamura K. I., Sano T. (2001). Case report: sinonasal teratocarcinosarcoma. *Pathology International*.

[24] Kurmi D. J., Mittal R. S., Sharma A., Gandhi A., Singhvi S. (2017). Sinonasal teratocarcinosarcoma involving nasal cavity, nasopharynx, and all paranasal sinuses with bilateral orbital and intracranial extension: a rare case report. *Asian Journal Neurosurg*.

[25] Agaimy A., Bishop J. A. (2021). SWI/SNF-deficient head and neck neoplasms: an overview. *Seminars in Diagnostic Pathology*.

[26] Chan-Penebre E., Armstrong K., Drew A. (2017). Selective killing of SMARCA2- and SMARCA4-deficient small cell carcinoma of the ovary, hypercalcemic type cells by inhibition of EZH2: in vitro and in vivo preclinical models. *Molecular Cancer Therapeutics*.

[27] Tagal V., Wei S., Zhang W. (2017). SMARCA4-inactivating mutations increase sensitivity to Aurora kinase A inhibitor VX-680 in non-small cell lung cancers. *Nature Communications*.

[28] Xue Y., Meehan B., Macdonald E. (2019). CDK4/6 inhibitors target SMARCA4-determined cyclin D1 deficiency in hypercalcemic small cell carcinoma of the ovary. *Nature Communications*.

[29] Minasi S., De Vincentiis L., D’Ecclesia A., Corsi A., Giangaspero F. (2021). Pathogenetic analysis of sinonasal teratocarcinosarcomas reveal actionable *β*-catenin overexpression and a *β*-catenin mutation. *Journal Neurol Surg B Skull Base*.

[30] Tchoyoson Lim C. C., Thiagarajan A., Sim C. S., Khoo M. L., Shakespeare T. P., Ng I. (2008). Craniospinal dissemination in teratocarcinosarcoma. *Journal of Neurosurgery*.

[31] Nguyen B. D. (2010). Sinonasal teratocarcinosarcoma: MRI and F18-FDG-PET/CT imaging. *Ear, Nose and Throat Journal*.

[32] Smith S. L., Hessel A. C., Luna M. A., Malpica A., Rosenthal D. I., El-Naggar A. K. (2008). Sinonasal teratocarcinosarcoma of the head and neck: a report of 10 patients treated at a single institution and comparison with reported series. *Archives of Otolaryngology- Head and Neck Surgery*.

[33] Modesto A., Blanchard P., Tao Y. G. (2013). Multimodal treatment and long-term outcome of patients with esthesioneuroblastoma. *Oral Oncology*.

[34] Amit M., Abdelmeguid A. S., Watcherporn T. (2019). Induction chemotherapy response as a guide for treatment optimization in sinonasal undifferentiated carcinoma. *Journal of Clinical Oncology*.

